# Association between inflammatory mediators and response to inhaled nitric oxide in a model of endotoxin-induced lung injury

**DOI:** 10.1186/cc7099

**Published:** 2008-10-27

**Authors:** Sebastien Trachsel, Ginette Deby-Dupont, Edwige Maurenbrecher, Monique Nys, Maurice Lamy, Göran Hedenstierna

**Affiliations:** 1 Department of Medical Sciences, Clinical Physiology, Uppsala University, S-75185 Uppsala, Sweden; 2 Department of Anesthesiology, University Hospital, Inselspital Bern, CH-3010 Bern, Switzerland; 3 Department of Anaesthesia and Intensive Care Medicine, University Hospital of Liège, Domaine du Sart Tilman – B35, B-4000, Liège, Belgium; 4 Centre for Oxygen Research and Development, Institute of Chemistry, B6a, University of Liège, Sart Tilman, Belgium University of Liège, B-4000 Liege, Belgium

## Abstract

**Introduction:**

Inhaled nitric oxide (INO) allows selective pulmonary vasodilation in acute respiratory distress syndrome and improves PaO_2 _by redistribution of pulmonary blood flow towards better ventilated parenchyma. One-third of patients are nonresponders to INO, however, and it is difficult to predict who will respond. The aim of the present study was to identify, within a panel of inflammatory mediators released during endotoxin-induced lung injury, specific mediators that are associated with a PaO_2 _response to INO.

**Methods:**

After animal ethics committee approval, pigs were anesthetized and exposed to 2 hours of endotoxin infusion. Levels of cytokines, prostanoid, leucotriene and endothelin-1 (ET-1) were sampled prior to endotoxin exposure and hourly thereafter. All animals were exposed to 40 ppm INO: 28 animals were exposed at either 4 hours or 6 hours and a subgroup of nine animals was exposed both at 4 hours and 6 hours after onset of endotoxin infusion.

**Results:**

Based on the response to INO, the animals were retrospectively placed into a responder group (increase in PaO_2 _≥ 20%) or a nonresponder group. All mediators increased with endotoxin infusion although no significant differences were seen between responders and nonresponders. There was a mean difference in ET-1, however, with lower levels in the nonresponder group than in the responder group, 0.1 pg/ml versus 3.0 pg/ml. Moreover, five animals in the group exposed twice to INO switched from responder to nonresponder and had decreased ET-1 levels (3.0 (2.5 to 7.5) pg/ml versus 0.1 (0.1 to 2.1) pg/ml, *P *< 0.05). The pulmonary artery pressure and ET-1 level were higher in future responders to INO.

**Conclusions:**

ET-1 may therefore be involved in mediating the response to INO.

## Introduction

Despite years of research and efforts for specific treatments of acute respiratory distress syndrome (ARDS), mortality remains significant [1]. A symptomatic approach aimed at fluid restriction, diuresis, reducing pulmonary hypertension and improving arterial oxygenation are the goals of therapy. The use of intravenous vasodilators to reduce pulmonary hypertension is limited because of deleterious side effects. Arterial oxygenation may worsen because of increased blood flow to nonventilated areas of the lung and systemic effects that can result in hypotension [2]. Inhaled nitric oxide (INO) allows selective pulmonary vasodilation and improves arterial oxygenation by redistribution of blood flow towards better ventilated parenchyma [3]. The clinical application of INO in ARDS and septic shock is still not definitive, however, and fails to show an improved outcome in ARDS [4-6]. Moreover, septic shock appears to be a condition associated with blunted response to INO [7] and nonresponse to INO occurs in about one-third of patients with ARDS [8].

Mechanisms of nonresponse to nitric oxide (NO) are proposed but remain inconclusive [9]. No indepth studies have been performed focusing on the systemic release of vasoactive inflammatory mediators and subsequent INO administration. We previously developed an experimental model of endotoxin infusion in pigs and tested the degree of response to INO 4 hours and 6 hours after onset of an endotoxin infusion [10]: a positive response, defined as a 20% PaO_2 _increase, was observed in most animals at 4 hours but not at 6 hours. This present report includes results from 28 animals. The aim of the study was to compare physiological and biochemical events to try to elucidate the mechanisms of response and nonresponse to INO in an endotoxin-induced animal lung injury model.

## Materials and methods

### Animals

After approval of the local Animal Research Ethical committee, 30 pathogen-free pigs (mixed Hampshire, Yorkshire and land race breeds) of either sex submitted to regular health testing were studied. Two pigs died before completion of the study, making a total of 28 pigs weighing 26.2 ± 1.0 kg.

### Experimental protocol

#### Anesthesia and catheterization

The protocol has been described previously [10]. After induction of anesthesia and tracheal intubation, mechanical ventilation (volume-cycled mode, Servo 900C; Siemens-Elema AB, Lund, Sweden) was performed with the following baseline settings: tidal volume, 10 ml/kg at 20 breaths/minute; inspiration to expiration ratio, 1:2; FiO_2_, 0.5; positive end-expiratory pressure, 5 cmH_2_O. The tidal volume was adjusted hourly to maintain normoventilation using the end-tidal carbon dioxide level as a guide (38.3 ± 0.5 mmHg).

Anesthesia was maintained by continuous infusion of clomethiazole (400 mg/hour, Heminevrin; Astra, Södertälje, Sweden), fentanyl (150 μg/hour) and pancuronium (2.5 mg/hour). Ringer acetate solution (1,000 ml; Pharmacia, Stockholm, Sweden) was infused before baseline measurements, in conditions to obtain a stable systemic pressure and a stable hemoglobin concentration (84 ± 1.1 g/l). Results on oxygenation are presented as the PaO_2_/FiO_2_.

A left carotid arterial line was inserted, and a Swan–Ganz catheter was introduced into the right jugular vein. The bladder was catheterized (balloon catheter Ch 20; Rüsch AG, Kernen, Germany) and peritoneal fluid drained via a multihole catheter.

#### Endotoxin infusion and nitric oxide challenges

After a stabilization period of 1 hour and baseline measurements, lung injury was induced by an endotoxin infusion (30 μg/kg/hour, *Escherichia coli *lipopolysaccharide 0111:B4; Sigma-Aldrich, Stockholm, Sweden) via a peripheral venous line over 2 hours. The animals were then given INO for a period of 10 minutes. A single exposure to INO was given to 20 animals at 4 hours and to another eight animals at 6 hours after onset of lung injury. Nine out of the 20 animals exposed to INO at 4 hours received a second INO challenge at 6 hours after the onset of lung injury, with the purpose of observing whether an animal changes its response to INO over time [10].

Nitric oxide (1,000 ppm; AGA Gas AB, Lidingö, Sweden) was delivered in an air/oxygen mixture from a low-flow air–oxygen blender (AGA AB, Sundbyberg, Sweden) into the low-pressure gas-flow inlet of the ventilator. The NO level was adjusted to an inspiratory concentration of 40 ppm, as measured by an NO chemiluminescence analyzer (9841 NOx; Lear Siegler Measurement Controls Corporation, Englewood, CO, USA). All measurements and blood gas sampling were collected after 10 minutes of NO inhalation. A positive response to INO was defined as a 20% increase in PaO_2 _compared with pretreatment levels [7].

At the end of the experiment the pigs were killed by an intravenous injection of potassium chloride (40 mmol).

#### Physiological parameters

The systemic mean arterial pressure, the mean pulmonary arterial pressure (MPAP) and the central venous pressure were continuously displayed and recorded (series 7010 Tram; Marquette Electronics, Milwaukee, WI, USA). The pulmonary capillary wedge pressure was measured intermittently, and the systemic vascular resistance and the pulmonary vascular resistance calculated. Cardiac output was determined by thermodilution using an injection of 8 ml cold 5% glucose solution. Arterial and mixed venous blood gases (oxygen partial pressure, carbon dioxide partial pressure, hemoglobin oxygen saturation), pH, and hemoglobin were analyzed by spectrophotometry with the analyzer calibrated for porcine blood (ABL 300 and OSM 3; Radiometer, Copenhagen, Denmark).

#### Blood sampling

Arterial blood samples were taken at baseline (T0) and every hour thereafter until 4 hours (T1 to T4) or 6 hours (T1 to T6); the blood samples at 4 hours and 6 hours were drawn just before NO inhalation. The total leukocyte count with the respective percentages of neutrophils and macrophages were obtained as well as the percentages of proteins, endotoxin (for control of the efficacy of the endotoxin infusion and evolution over time), cytokines (TNFα, IL-8), prostanoids (thromboxane B_2 _(TXB_2_), 6-keto-prostaglandin F 1 alpha (PGF_1α_) and prostaglandin F 2 alpha (PGF_2α_)), leucotriene B_4 _(LTB_4_), endothelin-1 (ET-1) and nitrates.

#### Biochemical parameters measurements

For endotoxin measurements, blood was drawn into pyrogen-free Chromogenix tubes and analyzed using a quantitative endpoint chromogenic method (Coatest; Chromogenix AB, Mölndal, Sweden). The *E. coli *0111:B4 reference endotoxin was the standard curve performed in pig serum or in sterile pyrogen-free water. The endotoxin value was expressed in picograms per milliliter and the lowest limit of detection was 5 pg/ml [11].

Cytokines (TNFα, IL-8) were measured in duplicate using commercially available cytokine-specific ELISA kits (Quantikine^®^; R&D Systems, Oxon, UK). The limits of sensitivity were 4.4 pg/ml for TNFα and 10 pg/ml for IL-8.

Prostanoids (TXB_2_, 6-keto-PGF_1α_, PGF_2α_) and LTB_4 _were measured by competitive enzyme immunoassay using commercially available kits (Cayman, Ann Arbor, MI, USA), after extraction on a C-18 reverse phase cartridge (Sep-Pak-C18 cartridges; Pharmacia). The limits of sensitivity were 13 pg/ml, 11 pg/ml, 8 pg/ml and 4 pg/ml for TXB_2_, 6-keto-PGF_1α_, PGF_2α _and LTB_4_, respectively.

ET-1 was measured by an immunometric assay using a commercially available kit (Cayman), after extraction on C-18 reverse phase cartridges. The limit of sensitivity was 1.5 pg/ml.

The kits used for cytokines and ET-1 measurements were valid for humans and pigs. The kits used for prostanoids and LTB_4 _are not species specific. Nitrates were measured by the Griess reaction in the presence of nitrate reductase. Proteins were measured by the Folin–Ciocalteu technique.

### Statistical analysis

Data are presented as the mean ± standard deviation or as the median (25th percentile to 75th percentile) when not normally distributed. One-way analysis of variance with Bonferroni correction was used for multiple comparisons. For comparison of two groups of values between responders and nonresponders or between two sampling times, we used the Wilcoxon test or the *t *test without correction for multiple comparisons. Comparisons between selected physiological and biochemical parameters not normally distributed were made by nonparametric correlation using the Spearman ρ coefficient (SPSS 14.0 for Windows; SPSS Inc., Chicago, IL, USA). Statistical significance was considered *P *< 0.05.

## Results

### Physiological events

There were no differences in any hemodynamic or gas exchange variable between 4 hours and 6 hours after induction of lung damage. The data for the single exposure to INO at 4 hours and 6 hours were therefore pooled for analysis.

#### Effect of endotoxin

Endotoxin exposure caused an increase in the MPAP and the pulmonary vascular resistance, whereas the cardiac output remained unaltered. There was a mean decrease in the mean arterial pressure. The systemic vascular resistance fell as well, but the decrease was only significant in nonresponders. Arterial oxygenation (PaO_2_/FiO_2_) was reduced and the PaCO_2 _increased with endotoxin infusion (Table 1).

**Table 1:** Hemodynamic parameters of responders and nonresponders

Parameter	Responder (n = 15)			Nonresponder (n = 13)		
	
	Baseline T0	Endotoxin	Inhaled nitric oxide	Baseline T0	Endotoxin	Inhaled nitric oxide
PaO_2_/FiO_2 _(mmHg)	502 ± 42	215 ± 118*	316 ± 141^†^	462 ± 62	234 ± 119*	225 ± 126
PaCO_2 _(mmHg)	37 ± 3.0	50 ± 11*	49 ± 11	39 ± 4.8	47 ± 7.1*	51 ± 9.2
pH	7.51 ± 0.03	7.29 ± 0.10*	7.22 ± 0.29	7.50 ± 0.04	7.34 ± 0.08*	7.30 ± 0.08
CO (l/min)	4.2 ± 1.2	4.4 ± 0.9	4.6 ± 1.2	4.1 ± 0.9	5.0 ± 1.5	5.4 ± 1.7
MPAP (mmHg)	16 ± 1.6	40 ± 7.7*	30 ± 7.5^†^	15 ± 2.3	33 ± 5.5* ^‡^	28 ± 5.0^†^
PVR (dyne/s/cm^5^)	222 ± 77	617 ± 231*	425 ± 179^†^	186 ± 38	407 ± 172* ^‡^	296 ± 125
CVP (mmHg)	4.0 ± 1.6	7.0 ± 3.0*	7.6 ± 3.9	4.8 ± 1.8	8.4 ± 3.2*	7.8 ± 2.8
MAP (mmHg)	85 ± 8.3	80 ± 17	77 ± 15	81 ± 9.8	78 ± 21	75 ± 22
SVR (dyne/s/cm^5^)	1,700 ± 436	1,380 ± 288	1,314 ± 341	1,541 ± 339	1,128 ± 256* ^‡^	1,032 ± 292^‡^
Qs/Qt (%)	9.4 ± 2.8^‡^	30 ± 20*	23 ± 17	12 ± 4.1^‡^	24 ± 9.0*	29 ± 12
Crs (ml/cmH_2_O)	27 ± 3.8	12 ± 3.2*	Not measured	26 ± 4.0	13 ± 3.2*	Not measured
Rrs (cmH_2_O·s/l)	15 ± 4.0	29 ± 8.8*	Not measured	15 ± 2.8	29 ± 8.6*	Not measured

Parameters were recorded at baseline T0, 4 hours after the start of endotoxin infusion, and after 15 minutes of nitric oxide inhalation. Data represent the mean ± standard deviation. CO, cardiac output; MPAP, mean pulmonary arterial pressure; PVR, pulmonary vascular resistance; CVP, central venous pressure; MAP, mean arterial pressure; SVR, systemic vascular resistance; Qs/Qt, intrapulmonary shunt; Crs, compliance of the respiratory system; Rrs, resistance of the respiratory system. *P < 0.05, endotoxin versus baseline; ^†^*P *< 0.05, inhaled nitric oxide versus endotoxin;^‡^*P *< 0.05, responder versus nonresponder.

#### Effect of inhaled nitric oxide

Inhalation of NO caused an increase in the PaO_2_/FiO_2 _of 50 mmHg (+22% of pre-INO PaO_2_/FiO_2_) when all pigs were pooled (n = 28). A decrease in the MPAP was seen with a mean of 8 mmHg (*P *< 0.05). There were 60% responders when data from all pigs were pooled. When the pigs were divided into a responder (20% increase in the PaO_2_/FiO_2_) and a nonresponder group, 65% of animals at 4 hours and 32% of animals at 6 hours after onset of endotoxin exposure were assigned the responder group. The PaO_2_/FiO_2 _increased from 215 mmHg to 316 mmHg in the responder group (*P *< 0.05), and the MPAP decreased from 40 mmHg to 30 mmHg (*P *< 0.05) (Table 1). The MPAP was significantly higher in the future responders and the decrease in MPAP during INO was twice as marked compared with the nonresponder group (Table 1). The venous admixture was reduced in the responder group during INO whereas it tended to increase in the nonresponder group (Table 1).

Of those nine animals exposed to a second NO challenge, seven pigs were responders at 4 hours and only two pigs were considered responders at 6 hours. Five animals had therefore become nonresponders at 6 hours after being considered responders at 4 hours. These five pigs increased the PaO_2_/FiO_2 _by 75% (*P *< 0.05) at the first exposure to INO but had no change in the PaO_2_/FiO_2 _at the second exposure.

### Blood cells and protein

#### Effect of endotoxin

The total leukocyte count decreased early, at T1 (1 hour after onset of endotoxin infusion) and on endotoxin exposure, and remained low until the end of the experiment (T4 to T6). Initially the neutrophils decreased as a fraction of the total leukocyte count, whereas macrophages increased. By the end of the experiment, the fraction of neutrophils had increased above baseline and macrophages were lowered. Platelets were decreased until T6. The blood protein concentration decreased until 3 hours after endotoxin administration and then remained low throughout the study period (Table 2).

**Table 2:** Cells, protein and inflammatory mediators from T0 (baseline) to T6

Parameter	T0 (n = 28)	T1 (n = **18**)	T2 (n = **18**)	T3 (n = **18**)	T4 (n = 28)	T5 (n = 9)	T6 (n = **9**)
Leucocytes (10^6^/ml)	9.38 ± 3.85	2.02 ± 0.5*	1.44 ± 0.89*	0.97 ± 0.19*	1.13 ± 0.49*	1.25 ± 0.41*	1.86 ± 0.79
Neutrophils (%)	53 ± 11	27 ± 10*	34 ± 8.9*	43 ± 15	54 ± 15	73 ± 16*	75 ± 11*
Macrophage (%)	46 ± 11	71 ± 11*	65 ± 9.3*	55 ± 15	44 ± 16	27 ± 17*	23 ± 11*
Thrombocytes (10^6^/ml)	384 ± 73	245 ± 51*	208 ± 48*	172 ± 41*	177 ± 55*	178 ± 65*	191 ± 52*
Proteins (mg/ml)	47 (42 to 52)	39 (34 to 46)*	33 (29 to 36)*	28 (23 to 34)*	35 (26 to 38)*	27 (22 to 31)*	34 (29 to 35)*
Endotoxin (pg/ml)	0 (0 to 0.04)	758 (542 to 1,008)*	783 (525 to 1,004)*	200 (125 to 755)*	85 (27 to 413)*	12 (5 to 73)*	2.3 (0.3 to 11)
Endothelin-1 (pg/ml)	0.1	5.0 (4.0 to 6.0)*	5.0 (5.0 to 6.5)*	5.0 (4.5 to 6.5)*	3.0 (2.0 to 7.5)*	4.5 (4 to 6.5)*	0.6 (0.1 to 8.8)*
PGF_2α _(pg/ml)	225 (167 to 344)	1,011 (859 to 1,216)*	1,301 (766 to 1,690)*	1,439 (971 to 1,708)*	387 (355 to 1513)*	1,028 (868 to 1,531)*	1,224 (333 to 1,562)*
TXB_2 _(pg/ml)	687 (581 to 793	3,110 (2,617 to 3,546)*	2,824 (2,120 to 3,287)*	2,409 (2,129 to 3,110)*	4,103 (1,521 to 4,986)*	2,107 (1,648 to 2,430)*	3,150 (891 to 4,002)*
6-keto-PGF_1α _(pg/ml)	294 (260 to 518)	749 (548 to 1778)	1,301 (899 to 1,658)*	732 (613 to 1341)	973 (716 to 1,504)*	464 (332 to 771)	877 (534 to 1,747)*
LTB_4 _(pg/ml)	36 (23 to 46)	42 (22 to 210)	69 (24 to 225)	48 (29 to 266)	52 (42 to 423)	70 (43 to 187)	240 (42 to 463)
TNFα (pg/ml)	17 (11 to 22)	53 (21 to 120)*	29 (5 to 52)	34 (14 to 69)	15 (10 to 38)	26 (0.5 to 60)	3.5 (2.0 to 9.5)
IL-8 (ng/ml)	0	2.0 (0 to 63)	117 (108 to 127)*	116 (104 to 126)*	31 (27 to 46)	39 (19 to 52)	6 (2 to 21)
Nitrates (nmol/ml)	211 (162 to 309)	151 (139 to 174)*	135 (114 to 172)*	133 (119 to 149)*	189 (147 to 237)	140 (120 to 157)*	190 (173 to 240)

Data presented as the mean ± standard deviation when normally distributed, or as the median (25th percentile to 75th percentile). PGF_2α_, prostaglandin F 2 alpha; TXB_2_, thromboxane B_2_; 6-keto-PGF_1α_, 6-keto-prostaglandin F 1 alpha; LTB_4_, leukotriene B_4_. *P < 0.05 when different to baseline.

#### Response to inhaled nitric oxide

In responders to INO the total leukocyte count and the fraction of neutrophils were higher compared with nonresponders (*P *< 0.05 for both comparisons) (Table 3). Thrombocytes and proteins were not different in the two groups.

**Table 3:** Cells and inflammatory parameters at baseline and before inhaled nitric oxide (INO) for responders and nonresponders

Parameter	Responders (n = 15)			Nonresponders (n = 13)		
	
	Baseline	Before INO	*P *value	Baseline	Before INO	*P *value
Leucocytes (10^6^/ml)	10.4 ± 4.4	1.6 ± 1.0^†^	≤ 0.01	8.8 ± 2.4	0.9 ± 0.3* ^†^	≤ 0.01
Neutrophils (%)	56 ± 12	65 ± 14	0.074	53 ± 10	48 ± 17*	0.45
Macrophage (%)	44 ± 12	34 ± 15	0.064	47 ± 10	51 ± 17*	0.45
Thrombocytes (10^6^/ml)	408 ± 77	185 ± 54^†^	≤ 0.01	355 ± 61	175 ± 68^†^	≤ 0.01
Proteins (mg/ml)	47 ± 6.0	33 ± 5.4^†^	≤ 0.01	46 ± 6	34 ± 5^†^	≤ 0.01
Endotoxin (pg/ml)	0	45 (3.8 to 296)^†^	≤ 0.01	0 (0 to 4.5)	32 (0 to 392)^†^	0.011
Endothelin-1 (pg/ml)	0	3.0 (1.8 to 8)^†^	≤ 0.01	0	0.1 (0.1 to 8)	0.31
PGF_2α _(pg/ml)	265 (236 to 327)	759 (356 to 1498)^†^	≤ 0.01	218 (171 to 512)	380 (342 to 1632)^†^	≤ 0.01
TXB_2 _(pg/ml)	692 (530 to 778)	2881 (860 to 4504)^†^	≤ 0.01	643 (574 to 821)	3883 (2286 to 5051)^†^	≤ 0.01
6-keto-PGF_1α _(pg/ml)	291 (232 to 323)	1023 (810 to 1560)^†^	≤ 0.01	518 (274 to 899)	603 (557 to 1099)	0.33
LTB_4 _(pg/ml)	28 (16 to 41)	372 (41 to 486)^†^	≤ 0.01	38 (9 to 48)	274 (46 to 414)^†^	≤ 0.01
TNFα (pg/ml)	15 (11 to 22)	18 (10 to 39)	0.083	12 (8 to 21)	10 (5 to 27)	1.0
IL-8 (ng/ml)	0	24(7.2 to 32)^†^	≤ 0.01	0	4 (1.5 to 47)^†^	0.03
Nitrates (nmol/ml)	212 (173 to 352)	183 (147 to 236)^†^	≤ 0.01	183 (133 to 255)	173 (150 to 212)	0.58

Data present as the mean ± standard deviation when normally distributed or as the median (25th percentile to 75th percentile). PGF_2α_, prostaglandin F 2 alpha; TXB_2_, thromboxane B_2_; 6-keto-PGF_1α_, 6-keto-prostaglandin F 1 alpha; LTB_4_, leukotriene B_4_. *P < 0.05 between responder and nonresponder, ^†^*P *< 0.05 between baseline and before INO.

### Biochemical variables

#### Effect of endotoxin

The endotoxin concentration in plasma increased after 1 hour of endotoxin infusion, slowly decreased after the cessation of the 2-hour infusion period and was no longer different from baseline at T6 (Table 2).

ET-1, TXB_2_, PGF_2α_, and TNFα all increased after 1 hour of endotoxin infusion and all remained elevated until the 6 hour measurement – except TNFα, which was no longer different from baseline at T2. 6-Keto-PGF_1α _and IL-8 differed from baseline after 2 hours of endotoxin infusion, and only 6-keto-PGF_1α _remained elevated at T4 and T6 (Table 2). The LTB_4 _levels did not differ from baseline.

The nitrate concentration in blood decreased initially but was not different from baseline in samples taken before the INO challenges at 4 hours and 6 hours (Table 2).

Positive correlation was seen between plasma concentrations of endotoxin and IL-8 (*ρ *= 0.62, *P *< 0.01). Further positive correlations were seen between MPAP on one hand and IL-8 (*ρ *= 0.72, *P *< 0.01) and ET-1 (*ρ *= 0.68, *P *< 0.01), on the other. Protein in plasma showed negative correlation with all parameters except 6-keto-PGF_1α _and TNFα.

#### Response to inhaled nitric oxide

**Figure 1:**
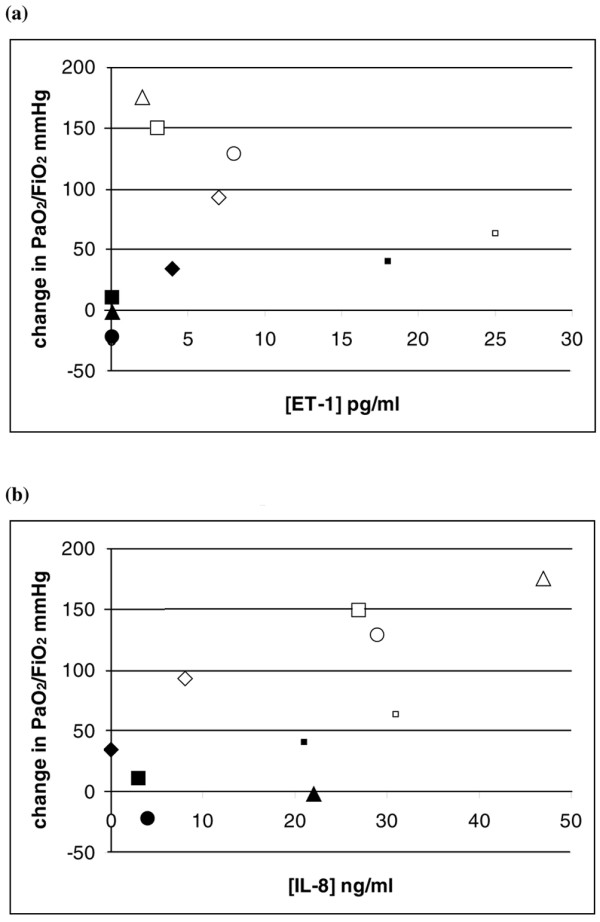
Levels of endothelin-1 and interleukin-8 compared with the increase or decrease of PaO_2_/FiO_2_. **(a) **Level of the endothelin-1 (ET-1) concentration (pg/ml) compared with the increase or decrease of PaO_2_/FiO_2 _(mmHg). All five animals exposed twice to inhaled nitric oxide decreased their ET-1 concentration level when changing from responder to nonresponder. **(b) **Level of IL-8 (ng/ml) compared with the increase or decrease of PaO_2_/FiO_2 _(mmHg). Levels of IL-8 decreased from response at the first exposure to nonresponse at the second exposure. Each symbol represents one animal; open symbol, first inhaled nitric oxide exposure; filled symbol, second exposure of the same animal with the same symbol.

The five animals that switched from being responders at 4 hours to become nonresponders at 6 hours showed less endothelin (3.0 (2.5 to 7.5) pg/ml versus 0.1 (0.1 to 2.1) pg/ml, *P *< 0.05) and less IL-8 (27 (16 to 28) ng/ml versus 1.5 (0 to 3.25) ng/ml;, *P *< 0.05) at the later occasion. The PaO_2_/FiO_2 _increase or decrease is plotted against the concentration of ET-1 and IL-8 in Figure 1a, b.

Less ET-1 in blood was seen in nonresponders in the total material (28 pigs) (0.1 (0.1 to 8) pg/ml versus 3.0 (1.8 to 8) pg/ml in responders), but this difference did not reach significance.

Prostanoids (PGF_2α_, TXB_2_, 6-keto-PGF_1α_) and LTB_4 _did not differ between responders and nonresponders, and neither did TNFα or nitrate concentrations (Table 3).

## Discussion

Endotoxin has dramatic and complex effects on the structure and function of the lungs in intact animals and also on isolated lung cells [12]. The 2-hour endotoxin infusion in the present model resulted in a marked lung dysfunction with a PaO_2_/FiO_2 _around 200 mmHg and pulmonary hypertension around 35 to 40 mmHg. Histological evidence of endotoxin-induced lung injury was previously performed and is described in a work by Da and colleagues [13]. The impairment remained stable over the 4-hour or 6-hour study period and 28 animals survived the whole experiment. Sixty percent of the animals were responders to a brief period of inhaled NO, similar to clinical observations in ARDS and sepsis [8].

The analysis of the animal group exposed twice to INO distinguished responders from nonresponders in terms of ET-1 and IL-8 levels. The responders had higher ET-1 and IL-8 levels.

### Inflammatory response to endotoxin

There was no difference in the endotoxin concentration in plasma at 4 hours between responders and nonresponders, but the rapid decrease in endotoxin concentration in the nonresponder group during the last 2-hour period suggests a faster metabolism of endotoxin in nonresponders. Endotoxin also binds to the endothelium, proteins and circulating cells, and this reduces the plasma concentration; however, whether this interacts with the vasodilating effect of inhaled NO is not clear.

The endotoxin infusion caused an early and severe leucopenia that may be explained by cell trapping and adhesion to the endothelium as well as dilution by edema formation. As shown earlier the fraction of neutrophils increased at the expense of the fraction of monocytes [10]. The higher neutrophil and macrophage count in responders than in nonresponders may illustrate a different inflammatory process due to the endotoxin infusion; however, this requires further study to be resolved.

Endotoxin also caused a rapid release of inflammatory mediators and vasoactive substances. Prostanoids, LTB_4_, ET-1, nitrates and cytokines increased in the blood. The vasoconstrictor TXB_2 _(a metabolite of thromboxane A_2_) also increased, and its concentration was always higher than that of the vasodilator 6-keto-PGF_1α _(metabolite of prostacycline). The levels of the proinflammatory cytokine IL-8 followed the endotoxin evolution in blood.

### Endothelin-1 and inhaled nitric oxide

The ET-1 in plasma rose rapidly and markedly already after 1 hour and paralleled the increase in blood endotoxin. There was a correlation between ET-1 levels and the pulmonary artery pressure. The higher MPAP in responders before INO challenge may therefore be explained by their higher ET-1 levels than in nonresponders. It may be argued that a general pulmonary vasoconstriction caused by an increased plasma concentration of ET-1 will facilitate or promote a positive response to INO and will improve oxygenation. This oxygenation improvement occurs because NO inhalation will cause vasodilation solely or preferentially in ventilated parenchyma, whereas the circulating ET-1 will promote vasoconstriction both in ventilated and nonventilated parenchyma. This mechanism may be comparable with the combination of almitrine with INO [14,15]. The stronger the pulmonary vasoconstriction, therefore, the more likely there will be a positive response to INO. This conclusion is also supported by the findings in the limited number of pigs exposed to INO at 4 hours and at 6 hours that switched from response to nonresponse, with lower ET-1 concentrations when no longer responding to INO. A continuous endotoxin infusion may have led to further responders since endothelin correlates with endotoxin levels [16]. The high initial dose of endotoxin (60 μg/kg) produced severe physiologic dysfunction that did not allow a prolongation of the endotoxin infusion.

It is still a matter of debate whether INO increases ET-1, which would accentuate vasoconstriction in nonventilated parenchyma [17]. In contrast, there is evidence that INO decreases ET-1 secretion [18]. Rebound hypertension after withdrawal from INO is attributed to ET-1 in an endotoxin lung injury model [19]. The role of ET-1 during INO may be selective vasoconstriction in nonventilated parenchyma, whereas ET-1 induces vasoconstriction in the entire pulmonary vascular bed after withdrawal of INO.

In isolated-perfused lungs from endotoxin-challenged rats, when nitric oxide synthase 2 is inhibited, responsiveness to INO improved [20]. This could be explained by the predominant vasoconstrictive effects induced by the suppression of endogenous NO. We were not able, however, to separate responders from nonresponders by different nitrate levels in this *in vivo *model.

### Severity of pulmonary damage

The severity of pulmonary dysfunction in terms of gas exchange and respiratory mechanics (compliance) did not differ between responders and nonresponders. The hemodynamics differed, however, with higher MPAP, pulmonary vascular resistance and systemic vascular resistance in the responders before INO challenge.

The degree of pulmonary damage separating responders from nonresponders may be an explanation for the varying responses to INO. Besides, a limitation may include the intravariability and intervariability of the animal lung injury model. On the contrary, most parameters of the inflammatory mediators measured here did not differ between responders and nonresponders – although IL-8 was, on an average, higher in the responder group [21]. This suggests that the severity of lung damage was much the same in the two groups. ET-1 and IL-8 were higher in responders, however, and ET-1 correlated to the MPAP. This observation may, as said above, explain higher values of the MPAP in responders. The separation between responders and nonresponders made on the basis of only two mediators (ET-1 and IL-8) may therefore support the hypothesis of a distinct mechanism, independent of the lung damage, responsible for the response to INO.

The results suggest that ET-1 may be a determining factor for a positive response to INO and for the decreased physiologic parameters. More severe pulmonary hypertension may be explained by higher levels of ET-1. INO, known for its antiinflammatory properties [22], could attenuate the effect of INO by decreasing ET-1 levels. This may explain the attenuation on oxygenation, when INO is administered for longer than 24 hours [4].

## Conclusion

The presented endotoxin lung injury model demonstrates that responders to INO present more severe pulmonary dysfunction at a comparable inflammatory profile. This observation can be explained by elevated ET-1 levels correlated to the magnitude of pulmonary hypertension that may result in a positive response to INO. This additionally supports the hypothesis that INO acts by two distinct mechanisms; one is vasodilation in ventilated lung regions, and the other is vasoconstriction in poorly ventilated or nonventilated lung regions. Other inflammatory parameters did not vary between responders and nonresponders, and possibly document similar injuries to the lung and its vasculature in the present study.

## Key messages

• Elevated concentration of endothelin-1 may mediate a positive response to inhaled nitric oxide.

• Responders to inhaled nitric oxide present more severe pulmonary dysfunction at a comparable inflammatory profile.

• Endothelin-1 levels correlate with the magnitude of pulmonary hypertension.

• Further support is added to the hypothesis that inhaled nitric oxide acts by two distinct mechanisms; one is vasodilation in ventilated lung regions, and the other is vasoconstriction in poorly ventilated or nonventilated lung regions.

## Abbreviations

ARDS: acute respiratory distress syndrome; ET-1: endothelin-1; IL: interleukin; INO: inhaled nitric oxide; 6-keto-PGF_1α_: 6-keto-prostaglandin F 1 alpha; LTB_4_: leukotriene B_4_; MPAP: mean pulmonary arterial pressure; NO: nitric oxide; PaCO_2_: arterial carbon dioxide partial pressure; PaO_2_: arterial oxygen partial pressure; PaO_2_/FiO_2_: ratio of arterial oxygen partial pressure to inspired oxygen fraction; PGF_2α_: prostaglandin F 2 alpha; TNFα: tumor necrosis factor alpha; TXB_2_: thromboxane B_2_.

## Competing interests

The authors declare that they have no competing interests.

## Authors' contributions

ST performed the statistical analysis and interpretation of data, edited the manuscript and acquired funding. GD-D was involved with the biochemical analysis (immunoassays) and with editing the manuscript. EM performed the experiments and was involved in data acquisition. MN participated in the biochemical analysis. ML was involved in the study design and in revising the manuscript. GH has made a substantial contribution to the design and conception of the study, and to the interpretation of data.
